# Exploring factors affecting individual GPS-based activity space and how researcher-defined food environments represent activity space, exposure and use of food outlets

**DOI:** 10.1186/s12942-021-00287-9

**Published:** 2021-07-28

**Authors:** Windi Lameck Marwa, Duncan Radley, Samantha Davis, James McKenna, Claire Griffiths

**Affiliations:** 1 Leeds Beckett University, Leeds, LS6 3QS UK; 2 Leeds Beckett University, Leeds, LS6 3QW UK; 3 Leeds Beckett University, City Campus, Leeds, LS1 3HE UK; 4 Leeds Beckett University, Headingley Campus, Leeds, LS6 3QS UK

**Keywords:** Global Positioning Systems, Activity space, Geographic Information System, Researcher-Defined Food Environments, Positive Predictive value, Sensitivity

## Abstract

**Background:**

Obesity remains one of the most challenging public health issues of our modern time. Despite the face validity of claims for influence, studies on the causes of obesity have reported the influence of the food environment to be inconsistent. This inconsistency has been attributed to the variability of measures used by researchers to represent the food environments—Researcher-Defined Food Environments (RDFE) like circular, street-network buffers, and others. This study (i.) determined an individual’s Activity Space (AS) (ii.) explored the accuracy of the RDFE in representing the AS, (iii.) investigated the accuracy of the RDFE in representing actual exposure, and (iv.) explored whether exposure to food outlet reflects the use of food outlets.

**Methods:**

Data were collected between June and December 2018. A total of 65 participants collected Global Positioning System (GPS) data, kept receipt of all their food purchases, completed a questionnaire about their personal information and had their weight and height measured. A buffer was created around the GPS points and merged to form an AS (GPS-based AS).

**Results:**

Statistical and geospatial analyses found that the AS size of participants working away from home was positively related to the Euclidean distance from home to workplace; the orientation (shape) of AS was also influenced by the direction of workplace from home and individual characteristics were not predictive of the size of AS. Consistent with some previous studies, all types and sizes of RDFE variably misrepresented individual exposure in the food environments. Importantly, the accuracy of the RDFE was significantly improved by including both the home and workplace domains. The study also found no correlation between exposure and use of food outlets.

**Conclusions:**

Home and workplace are key activity nodes in modelling AS or food environments and the relationship between exposure and use is more complex than is currently suggested in both empirical and policy literature.

## Background

Obesity remains one of the most challenging public health issues globally and its prevalence is still rising [44, 60]. One of the factors that could have an important role in the current obesity epidemic is the environment [39, 45]. In the past five decades, the developed nations have experienced a change in their food environments characterised by increased access to energy-dense food [2, 23, 51]. Although the concept of environmental influence on obesity is appealing, the evidence to support it is still inconsistent [9, 63].

The inconsistency in the link between the food environment and health outcomes could be attributed to not only individual-level variability in the dose–response relationship but also the variation of measures used to represent food environments [63]. The evidence on the dose–response relationship between exposure to food and dietary intake is limited. The dearth of knowledge in this area is partly contributed to by intricate and intertwined social, temporal and spatial aspects of the interaction between people and place, making the effective measure of exposure challenging [12].

A body of evidence in the food environment field is underpinned by research using fixed point-based anchors such as home, school or workplace and area-based anchors such as census tract, electoral wards, postcode, or other administrative units [9, 63]. Due to the increased availability of secondary data containing individuals’ geographic information like individuals’ home and workplace, there has been an increase in studies examining environmental influences of health using fixed point-based anchors measures of physical environments in which different buffer types and sizes were created around fixed points (i.e. homes or workplaces) to represent individuals’ food environments. In this study, spaces created by researchers around participants home, workplace and en route were referred to as Researcher-Defined Food Environments (RDFE). Cummins et al. [11] refer to this approach as a conventional view of the food environment, whereby places are geographically defined space with boundaries identified at a specific scale spaced by a physical distance (e.g. zip codes, census tract and postal codes) [11]. Despite being commonly used by researchers in food environment research, this approach has been critiqued for minimising the size of the environment and exposure [11, 65].

Cummins et al. [11] suggest an alternative view of environment—a relational view. This view considers points of interest (places) as nodes in networks rather than discrete and autonomous bounded spatial units which are unstructured, unbounded and freely connected with human practice forming connection patterns [11, 38]. Hudson [25] reiterates this view when he describes these nodes and networks as complex circuitry with multiple linkages and feedback loops. The advent of technologies like Global Positioning System (GPS) has provided effective means of capturing individual Activity Space (AS)—a relational view of the environment—which represents opportunities for the regular and daily experience of people in multiple settings. AS refers to “the area within which people move or travel in the course of their daily activities” [40], p.439). Likely, AS can stretch beyond the residential neighbourhood, incorporating a holistic range of exposures.

Although AS has been examined by various health researchers [13, 37, 43, 47, 52, 56], Weerdenburg, van et al. 2019; [64], only a handful of studies have used it to explore the influence of food environment on individuals’ health. Several studies have found that area of AS bigger than RDFE. Zenk et al. [65] for example found in their study that AS were bigger than home neighbourhoods and dietary behaviours had a statistically significant link to AS environmental characteristics. Likewise, Crawford et al. [10] found that the average area for participant-defined neighbourhoods (0.04 square miles) was smaller (2 miles) compared to the road network neighbourhoods (3 square miles) and AS (26 square miles). They also noted in their study that AS provided the greatest exposure than other measures. Food environment refers to all opportunities for someone to obtain food, including physical, socio-cultural, economic and policy factors at micro-level (local settings such as schools, homes, and workplaces) and macro-level (broader environments or sectors like education, health systems and food industry) [31, 54].

Furthermore, it is suggested that individual’s sociodemographic and socioeconomic characteristics (age, gender, educational attainment, occupation and income) and environmental factors (e.g. residential place and land use) characteristics could have an influence on the nature and characteristics of an individual’s daily mobility [29]. A study by Widener et al. [61] for instance showed that individuals demographics, household food shopper status and city of residence had a significant association with different levels of exposure to various food outlets. Also, food shopping behaviours were statistically significantly associated with demographics, the activity space-based food environment, self-reported health and city of residence. Despite these findings, there is a limited research on the influence of individual and environmental characteristics on an individual’s AS overall.

When considering exposure, a study by Burgoine et al. [6] revealed that exposure to takeaway food outlets in the home, work, and commuting environments combined was linked to marginally higher consumption of takeaway food, greater body mass index, and greater odds of obesity. Similarly, a study by Sadler et al. [49] in adolescents showed that the exposure to ‘unhealthy food outlets’ between home and school had a significantly increased likelihood of purchasing junk food. The downside of these studies is that they did not explore the relationship of the exposure and use of food outlets, nor whether the stores classified as ‘unhealthy’ were the major contributor source of the increased purchase and consumption of junk food. It is worth noting that the so called “healthy” food outlets like supermarkets and “unhealthy” food outlets like convenience stores can sell or serve both healthy and unhealthy food options or portions [27]. This study set out to i) determine an individual’s AS and explore both individual and environmental factors influencing AS, ii) examine how RDFE represent AS, iii) investigate the accuracy of RDFE in representing actual exposure, and iv) whether exposure to food outlets is associated with use.

## Methods

This study was undertaken between June and December 2018. It included participants residing in Leeds, UK and its surrounding areas, aged 18 years old and above, with proficient digital skills (e.g. smartphone, laptop or computer) and access to the internet. A non-representative volunteer sample of 76 participants enrolled for this study through poster advertisements in Leeds UK. Three participants withdrew before data collection and eight participants were excluded due to errors in their GPS data resulting in a final sample of 65. The study received ethical approval from Leeds Beckett University Research Ethics Committee (ref. 37629).

### Individual characteristics data

Participants completed an online questionnaire via Qualtrics to capture their socio-demographic and socio-economic status (SES) characteristics, including age, gender, occupation, income, residential postcodes and workplace addresses. Note, home addresses were considered as sensitive information and not collected. Residential postcodes were used to generate Index of Multiple Deprivation (IMD) ranks using an online IMD postcode lookup (http://imd-by-postcode.opendatacommunities.org/) and grouped into IMD quintiles. Home postcodes were geocoded to postcode zone centroids to protect participants identity while workplace and food purchase locations were geocoded at the address level. The geocoding was done using ArcGIS. Participants weights (kg) and heights (m) were measured, and participants’ Body Mass Index (BMI) used to classify them as ‘underweight’ (BMI < 18.5 kg/m^2^) ‘healthy’ (BMI = 18.5–24.9 kg/m^2^), ‘overweight’ (BMI = 25–29.9 kg/m^2^) or ‘obese’ (BMI ≥ 30 kg/m^2^).

### Food purchase location and dietary data

For seven days, participants were asked to keep receipts of all their food purchases. The receipts provided addresses of food outlets visited, date and food items purchased. If participants forgot to collect receipts or food outlets did not provide receipts, participants were asked to record the name of the food outlet, date and food items purchased on a piece of paper whenever possible. The addresses of food venues were geocoded (converted into XY coordinates) and used in geospatial analysis while other information such as type and amount of food was used for validation of dietary intake.

### Activity space

Participants were issued GPS tracking devices and asked to wear them every time they moved outside their residential places for a period of seven days including week and weekend days. The study used Garmin Foretrex 301 and 401 devices which were set to record GPS points every 30 s. The collected GPS data were used to create a buffer of 1 km on both sides of the road network which was considered as an AS (GPS-based AS). This buffer size was considered a reasonable estimate of how far a person would walk [28, 65] and captures the adjacent area to individual daily paths. According to Li and Tong [35], ‘Activity spaces are geographical measures of the locations, paths, and areas adjacent to where people go to carry out their daily lives’. Christian [8], Sadler and Gilliland [48] and Zenk et al. [65] for example created 0.5-mile (0.8 km) around all GPS points and dissolved them into a single feature or space to represent the adjacent areas around participants’ daily path. Likewise Sherman et al. [52] and Kerr et al. [28] used 1 km buffers in their studies. A systematic review by Smith et al*.* [53] also included several studies that used daily path areas—buffers of all points or tracks.

### Researcher-defined food environments (RDFE)

In this study, Researcher-Defined Food Environments (RDFE) refer to different types of buffers (e.g. circular and street network buffers) frequently used by researchers as a measure of an individual’s food environment. A variety of RDFE were created using three buffer sizes 2 km, 4 km and 6 km. These buffer sizes have been used in previous studies and in this study they were used to allow comparisons [24, 28]. Table 1 shows graphical representations of each RDFE.

**Table 1:** Definition of terms

Buffer type	Shape	Definition
Activity space (AS) [Calculated using GPS data]		An area within which individual moves or travels during the course of their daily activities (i.e. everywhere a participant went for 7 days)
Straight-line buffer (SLB) [Calculated using questionnaire data]		An area around a straight-line distance between two points of interest [18] (i.e. a buffer around a Euclidean distance between participant’s home and workplace)
Circular buffer around home/workplace (CBH/W) [Calculated using questionnaire data]		A circular area around home (i.e. a circular buffer around a participant’s home/workplace)
Circular buffer around both home and workplace (CBHW) [Calculated using questionnaire data]		An area formed by two circular buffers around home and workplace. It can be fused or two distinct circular areas
Street network buffer around workplace (SNBH/W) [Calculated using questionnaire data]		An area that could be reached by a participant within a specified travel distance (i.e. 2 km, 4 km and 6 km) from home/workplace along a street network
Standard Deviational Ellipse (Lobstein et al*.*) [Calculated using GPS data]		A measure of the trend of points (i.e. GPS points of participant’s movements) or orientation of an area (i.e. AS) 2 SDE refers to space around 95% of all GPS points of participants movements

**Fig. 1:**
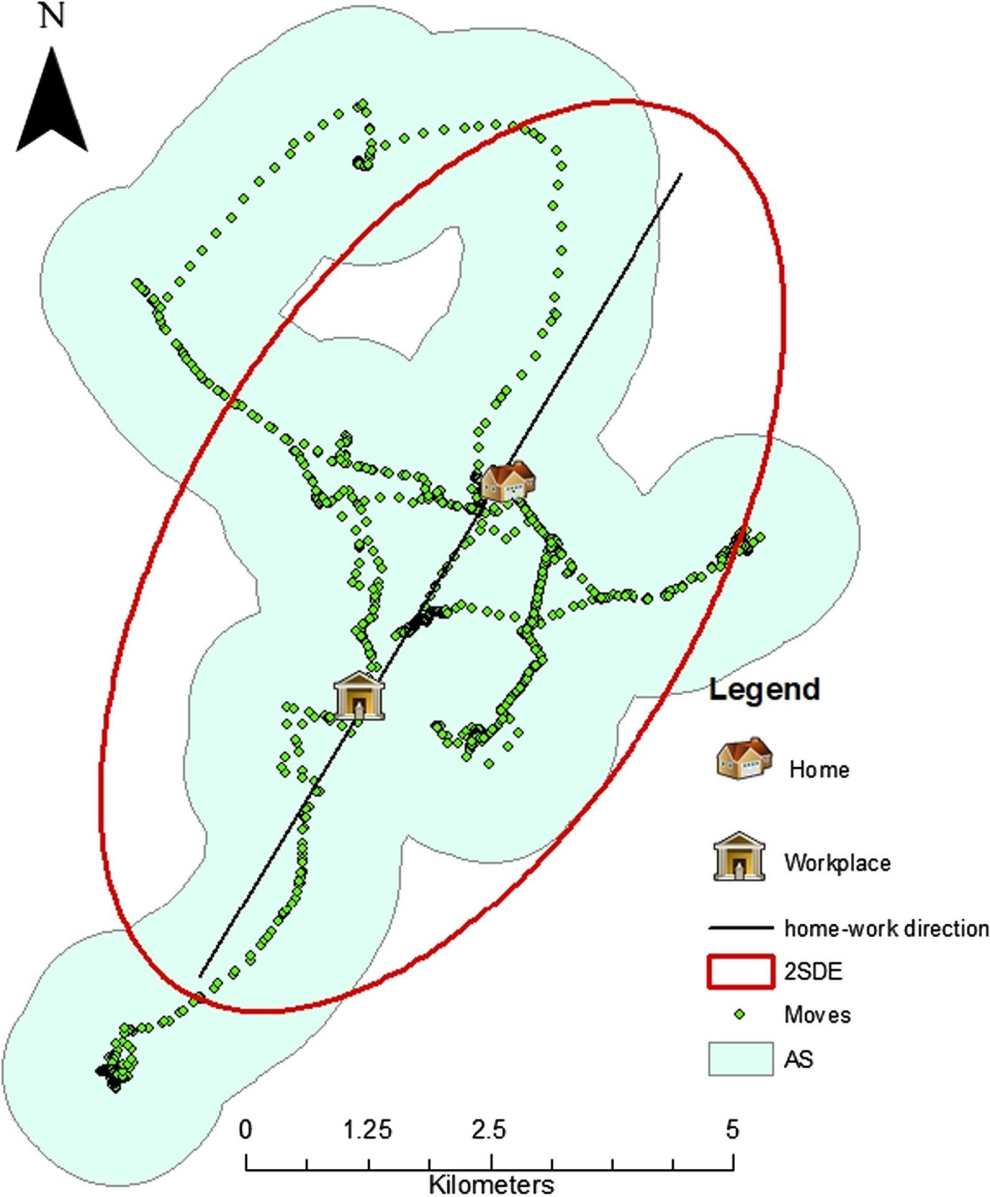
Example of 2 SDE orientation

Standard Deviational Ellipses (SDE) were also created to determine the dispersion of GPS points (participant’s movements) and the orientation of participants’ mobility and AS. According to Wang et al*.* [58], SDE are useful in identifying a dispersion or concentration and orientation of spatial features. In this study, 2 SDE were created to capture 95% of all GPS points of participants movements. The 2 SDE were used to assess any pattern on participants’ movements that could influence the shape (orientation) of their AS. Using visual inspection, the orientations of 2 SDE were assessed for agreement with home-workplace direction and recorded as a binary variable (yes/no) (see Fig. 1).

### Points of interest and road-network data

This research also used PoI and Road-Network (RN) data of 2018. The PoI data set was used to identify and map all food outlets in the study area. The PoI data were obtained from the Edina Digimap website (http://digimap.edina.ac.uk). The PoI features codes were decoded into features names and cleaned to remove all non-food outlet features. The features retained in the PoI data included supermarkets, convenience stores, fast-food outlets, and others (i.e. cafés and coffee shops, speciality food stores, pubs and inns, grocery stores, hotels and restaurants). Identical outlets were identified and removed resulting in a final sample of 20,306 outlets. The PoI data has been validated and showed to be accurate for classifying food outlets [62].

The RN data provided comprehensive data on all major and minor roads except private roads in the study area. The RN data were also downloaded from the Edina Digimap website (http://digimap.edina.ac.uk) and converted into a road network dataset before data analysis.

### Data analysis

Statistical and geospatial analyses were undertaken in SPSS version 25 and ArcGIS 10.2, respectively. Tests of normality were conducted to guide a choice of the appropriate test for the analysis. The assumption of normality for AS (continuous variables) was not satisfied as assessed by Shapiro–Wilk's test (p > 0.05) and by visual inspection of Normal Q-Q Plots. Due to violation of normality assumption, non-parametric tests Mann–Whitney U and Kruskal–Wallis H were used to examine mean differences of between groups of binary variables and Kruskal–Wallis H was used for variables with more than 2 categories. Subsequent pairwise comparisons with Bonferroni adjustment for multiple comparisons were used to assess the difference in AS between groups. Multiple linear regression analysis was performed to assess the relationships between participants’ SES, sociodemographic characteristics, Euclidean distance from home to workplace and AS size. Overlap between RDFE and AS was calculated in ArcGIS as a proportion of intersection and presented as mean percentage overlaps and analysed further in SPSS to determine the agreement between RDFE and AS. The Wilcoxon paired rank test was used to explore differences in sizes of food environment captured by the RDFE and AS.

**Fig. 2:**
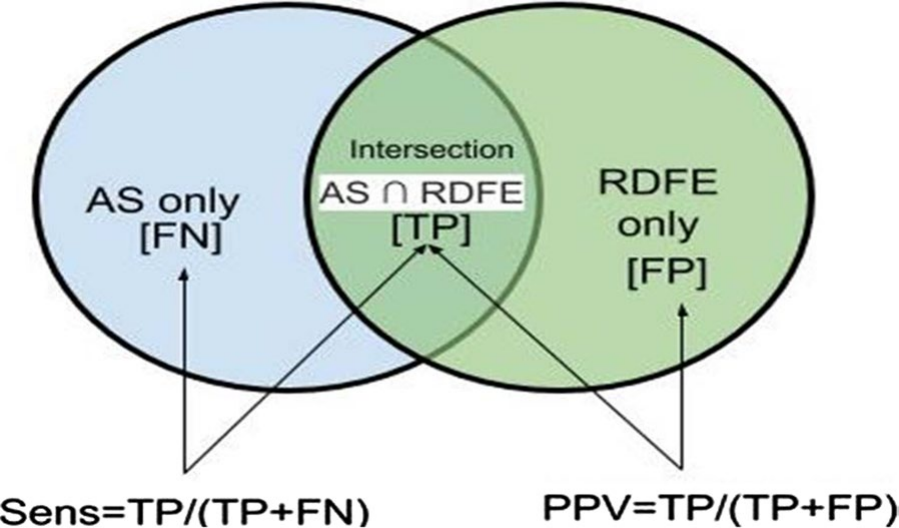
An illustration of the intersection between an AS and RDFE Key: *AS*  Activity Space, *RDFE*   Researcher-Defined Food Environments, *Sens*   Sensitivity, *TP*   true Positive, *FN*   False Negative,* FP*  False Positive.

The accuracy of the RDFE to represent exposure was assessed using Positive Predictive Value (PPV) and sensitivity. PPV is the proportion of positive results that are true positives, calculated as True Positive [TP] / (True Positive [TP] + False Positive [FP]) [17]. The PPV in this study denoted the proportion of food outlets within the RDFE that were truly present in an individual’s AS. The TP value represented the outlets in the AS that were correctly captured by RDFE and the FP value denoted the outlets captured by RDFE which were not within the AS (Fig. 2). Sensitivity on the other hand measures the proportion of actual positive results that are correctly identified by a measure, calculated as True Positive [TP] / (True Positive [TP] + False Negative [FN]) (Fletcher et al. 2012). FN denoted the outlets within the AS which were missed by RDFE (Fig. 2). The value of both PPV and sensitivity range from 0 to 1 (i.e. 0–100%).

In this study, exposure referred to food outlets within a defined environment and use referred to the food outlets in which the participants purchased food.

## Results

Table 2 displays the full characteristics of the sample. Participants were aged between 19 and 67 years old (mean = 37 ± 13 years) (Table 2). The majority of participants were female (72%) which could be because study participation was voluntary and the study could have more appeal to women than men. This is not uncommon in health promotion research and programmes in which men tend to have lower participation than female overall [36, 42, 46]. More than half of the participants (60%) were White, nearly three-quarters (71%) were educated to degree level or higher, and 78% were in full-time employment. Most of the participants (92%) worked away from home and 60% used a car to commute from home to their workplace. Likewise, the high proportion of highly educated and full-time employed participants could be due to the voluntary participation in the study and the appeal of the study to these groups. More than half of the participants (63%) had a healthy weight. The BMI of participants ranged from 18 to 37 kg/m^2^ with a mean BMI of 25 kg/m^2^.

**Table 2:** Participants’ characteristics

Characteristics	n (%)
Gender	
Female	47 (72)
Male	18 (27)
Age group (years)	
18–20	11 (17)
21–30	12 (18)
31–40	16 (25)
41–50	16 (25)
> 50	10 (15)
Ethnicity	
White	39 (60)
Non-white	26 (40)
Education attainment	
A Levels	16 (23)
Diploma	4 (6)
Degree	20 (31)
Postgraduate degree	26 (40)
Place of work	
Away from home	60 (92)
Home	5 (8)
Transport mode from home to workplace	
Walking	12 (18)
Bus	9 (14)
Car	39 (60)
Bicycle	2 (3)
Train	3 (5)
Personal annual income	
Under £10,000	14 (22)
£10,000—< £25,000	22 (34)
£25,000—< £50,000	24 (37)
£50,000—< £100,000	3 (5)
£100,000 and more	1 (2)
Household annual income	
Under £10,000	11 (17)
£10,000–< £25,000	10 (15)
£25,0000–< £50,000	27 (42)
£50,000–< £100,000	15 (23)
£100,000 +	2 (3)
Working hours/day	
Under 8 h	28 (43)
8 h to less than 12	31 (48)
12 h and more	6 (9)
IMD quintile	
1 (most deprived)	5 (8)
2	19 (29)
3	15 (23)
4	10 (15)
5 (least deprived)	16 (25)
Weight status	
Underweight	0 (0)
Healthy	41 (63)
Overweight	14 (22)
Obese	10 (15)
Occupational social class	
Managerial and professional	33 (51)
Intermediate	11 (17)
Routine and manual	21 (32)
Age (years) Mean = 37, [Min = 19; Max = 67], SD = 13	
BMI (kg/m^2^) Mean = 25, [Min = 18; Max = 37], SD = 5	
AS (km^2^) Mean = 62, [Min = 6; Max = 284], SD = 58	
Euclidean distance (home-workplace) Mean = 5, [Min = 0, Max = 21], SD = 5	

### Aim 1: Determine an individual’s AS and explore both individual and environmental factors influencing AS

**Fig. 3:**
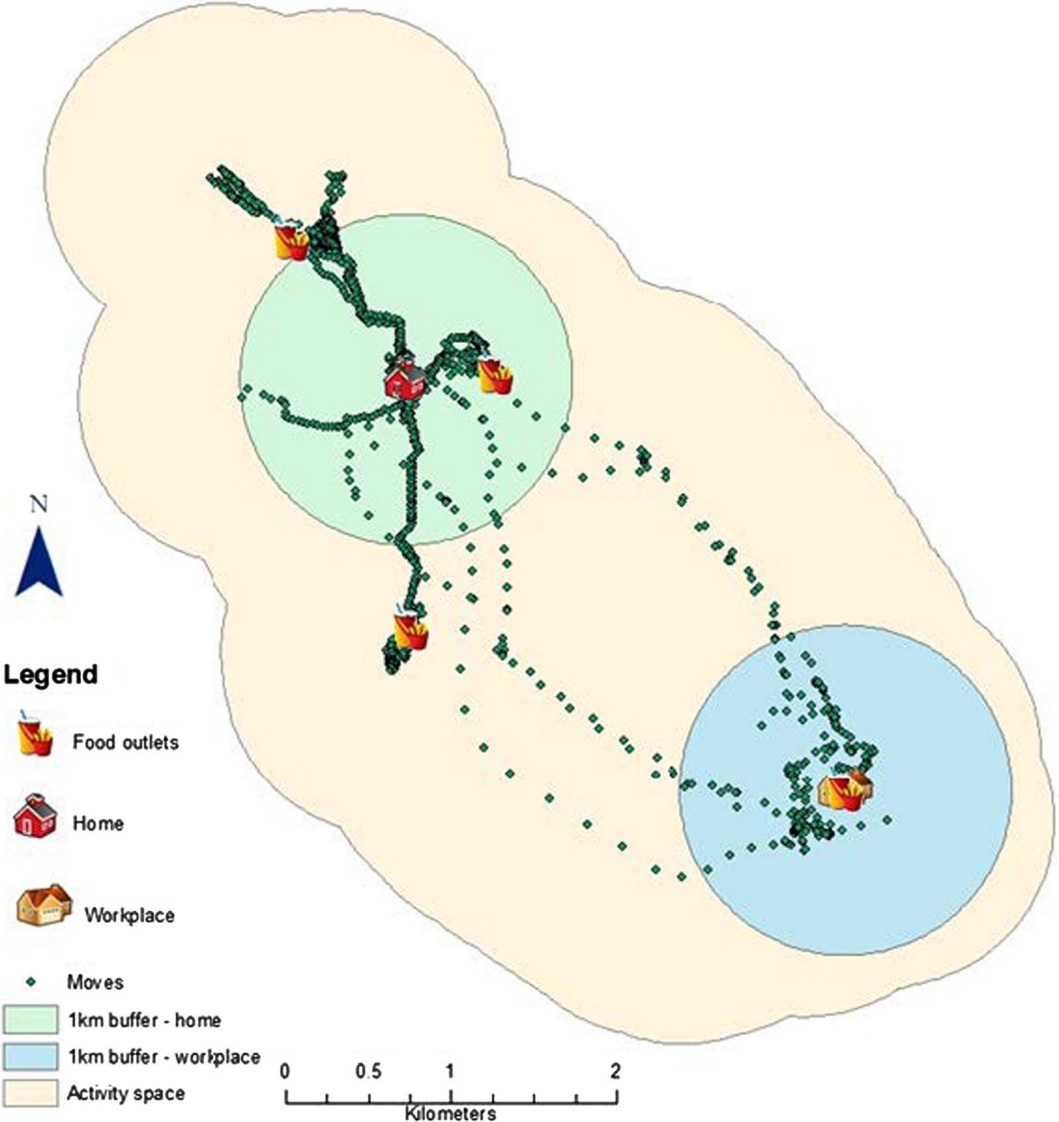
An example of the concentration of mobility between home and workplace for a participant working away from home

The average AS size was 62 (min = 6, max = 284 km^2^), and Euclidean distance from home to workplace 5 km (min = 0, max = 21 km) (Table 1). Most movements of participants working away from home were between home and workplace (Fig. 3).

Assessment of the orientation of AS using 2 SDE of participants’ movements revealed that the AS of 60/65 (82%) of participants who worked away from home followed home-to-workplace direction and most of the concentration of GPS points for these participants was in the space between home and workplace (Fig. 3).

The Mann–Whitney U test showed that median AS size was statistically significantly smaller in individuals using active transport (walking and cycling) than in individuals using motorised transport (car, bus, and train) (p = 0.011). No statistically significant difference in AS size was detected between gender (p = 0.482), employment status (p = 0.285), shifts (p = 0.146) and working hours (p = 0.165) groups (Table 3). The Kruskal–Wallis test showed statistically significant difference in AS between the different age groups, **χ**^*2*^ (4) = 18.77, *p* = 0.001. The post hoc analysis showed statistically significant differences in median AS between participants aged 18–20 years and 41–50 years (*p* =  < 0.001) as well as 21–30 years and 41–50 years (*p* = 0.002). (Table 3).

**Table 3:** Differences in AS according to participants’ characteristics

Characteristics	n	Mean Rank	U	z	p-value
Mann–Whitney U test					
Gender					
Female	47	34	375	− 0.7	0.482
Male	18	30			
Employment status					
Full-time	51	34	290	− 1.07	0.285
Part-time	14	28			
Work shift					
No	54	34	214	− 1.45	0.146
Yes	11	26			
Working hours					
≤ 8 h	51	31	444	1.39	0.165
> 8 h	14	39			
Transport mode from home to workplace					
Active	15	22	539	2.55	0.011*
Motorised	50	36			

Characteristics	n	Mean Rank	**χ**^*2*^	df	p-value
Kruskal–Wallis Test					
Age group (years)					
18–20	11	19	18.77	4	0.001
21–30	12	25			
31–40	16	36			
41–50	16	48			
> 50	10	30			
IMD quintile					
1	5	37	6.7	4	0.152
2	19	25			
3	15	31			
4	10	36			
5	16	41			

*p-value < 0.05; **χ**^*2*^ = Chi-squire

Regression analysis showed that for every 1 km increase in the Euclidean distance from home to work there was an increase of 34.04km^2^ of AS (CI:13.19, 54.90, p = 0.002). No individual characteristics were predictive of individual AS size (Table 4).

**Table 4:** Regression analysis results exploring the relationship between sociodemographic and socioeconomic characteristics and AS size

	β	95%CI		p-value
		Upper	Lower	
(Constant)	− 19.56	− 164.31	125.19	0.786
Gender	31.12	− 0.1	62.14	0.063
Age	− 0.17	− 1.79	1.46	0.837
Ethnicity	− 17.73	− 35.32	0.15	0.061
Level of education	9.95	− 3.03	22.93	0.130
Employment status	− 1.22	− 33.5	31.06	0.939
Shift	13.5	− 24.06	51.06	0.472
Working hours	− 2.83	− 25.95	20.29	0.806
IMD quintile	3.78	− 18.19	25.76	0.730
BMI	2.45	− 4.19	9.08	0.461
Weight status	− 8.88	− 44.8	27.04	0.620
Occupational social class	− 7.1	− 32.32	18.11	0.573
Personal annual income	13.85	− 13.56	41.25	0.314
Household annual income	− 10.54	− 35.06	13.98	0.391
Transport mode	− 26.26	− 62.44	9.92	0.150
Euclidean distance (home-workplace)	34.04	13.19	54.9	0.002*

*AS * dependent variable, *IMD*  Index of Multiple deprivation, *BMI  *Body Mass Index

*p-value < 0.05

### Aim 2: Examine how RDFE represent AS

All RDFE explored in this study had limited accuracy in representing AS (Table 5). The mean percentage overlap between RDFE and AS varied according to the type and size of the buffer used. For instance, small buffers of 2 km had very low percentage mean overlap such as CBH (16.6%), CBW (14.6%), CBHW (27.5%), SNBH (8.5%), SNBW (7.8%) and SLB (40.7%). The percentage mean overlap showed a direct relationship with the buffer size, meaning that the percentage mean overlap increased as the buffer size increased. To note, circular buffers around the home or workplace alone underestimated AS by more than 50%. Underestimation was less severe for buffers involving both home and workplace (e.g., CBHW and SLB) but was still significant.

**Table 5:** Overlap between RDFE and AS

RDFE		Mean AS (km^2^)	Mean overlap (km^2^)	% mean overlap	Mean difference	95% CI for difference		% mean difference	p-value
Type	Size					Lower	Upper		
CBH	2 km	59.54	9.87	16.6	49.67	36.09	63.27	83.4	< 0.001*
	4 km	59.54	21.96	36.9	37.58	24.65	50.51	63.1	< 0.001*
	6 km	59.54	31.07	52.2	28.47	16.78	40.16	47.8	< 0.001*
CBW	2 km	59.54	8.66	14.5	50.88	37.18	64.59	85.5	< 0.001*
	4 km	59.54	19.08	32.0	40.46	27.34	53.59	68.0	< 0.001*
	6 km	59.54	28.88	48.5	30.66	18.76	42.58	51.5	< 0.001*
CBHW	2 km	59.54	16.35	27.5	43.19	29.94	56.45	72.5	< 0.001*
	4 km	59.54	31.42	52.8	28.12	16.48	39.78	47.2	< 0.001*
	6 km	59.54	40.61	68.2	18.93	9.59	28.27	31.8	< 0.001*
SNBH	2 km	59.54	5.08	8.5	54.46	40.75	68.18	91.5	< 0.001*
	4 km	59.54	15.22	25.6	44.32	30.79	57.85	74.4	< 0.001*
	6 km	59.54	24.07	40.4	35.47	22.65	48.3	59.6	< 0.001*
SNBW	2 km	59.54	4.67	7.8	54.87	41.26	68.49	92.2	< 0.001*
	4 km	59.54	13.84	23.2	45.70	32.32	59.09	76.8	< 0.001*
	6 km	59.54	22.65	38.0	36.89	24.21	49.58	62.0	< 0.001*
SLB	2 km	59.54	24.24	40.7	35.30	23.54	47.08	59.3	< 0.001*
	4 km	59.54	36.66	61.6	22.88	12.79	32.97	38.4	< 0.001*
	6 km	59.54	35.47	73.1	16.01	7.61	24.4	26.9	< 0.001*
2 SDE		59.54	35.47	59.6	24.07	18.41	29.74	40.4	< 0.001*

N = 60, *RDFE*  Researcher-Defined Food Environment, *AS * Activity space *CBH * Circular Buffer around Home, *CBW*  Circular Buffer around Workplace, *SNBH*  Street Network Buffer around Home, *SNBW*  Street Network around Workplace, *SLB*  Straight-line Buffer, *2 SDE*   2 Standard Deviational Ellipse. Note, CBHW is a union of circular buffers around home and workplace and not a sum of individual circular buffers

### Aim 3: Investigate the accuracy of RDFE in representing individual exposure to food outlets

In all food environments (i.e. AS and RDFE), ‘other’ food outlets were the majority, followed by fast-food outlets. Supermarkets had the lowest count in all food environments. The average participant had access to 621 (min = 63, max = 1170) ‘other’ food outlets, 299 (min = 42, max = 654) fast-food outlet, 231 (min = 15, max = 555) convenience stores and 27 (min = 2, max = 73) supermarkets within their AS. A similar distribution was evident in all RDFE (Table 6).

**Table 6:** Food outlet count within AS and RDFE

Food environment metric	Size	Food outlet type	(Min, Max)	Mean	SD
AS		Convenience store	(15, 555)	231	130
		Fast-food Outlet	(42, 654)	299	151
		Supermarket	(2, 73)	27	15
		Other Food Outlet	(63, 1170)	621	341
Circular buffer around home	2 km	Convenience store	(0, 131)	32	27
		Fast-food Outlet	(1, 206)	63	50
		Supermarket	(0, 15)	7	4
		Other Food Outlet	(4, 616)	87	108
	4 km	Convenience store	(3, 288)	113	69
		Fast-food Outlet	(5, 514)	212	133
		Supermarket	(0, 45)	22	11
		Other Food Outlet	(11, 935)	337	281
	6 km	Convenience store	(9, 378)	219	105
		Fast-food Outlet	(15, 691)	406	193
		Supermarket	(2, 75)	42	18
		Other Food Outlet	(32, 1152)	682	368
Circular buffer around workplace	2 km	Convenience store	(2, 120)	44	36
		Fast-food Outlet	(4, 232)	88	64
		Supermarket	(1, 14)	7	3
	4 km	Convenience store	(3, 295)	156	74
		Fast-food Outlet	(6, 529)	293	127
		Supermarket	(2, 46)	28	9
		Other Food Outlet	(21, 944)	502	309
	6 km	Convenience store	(12, 375)	264	91
		Fast-food Outlet	(26, 652)	481	154
		Supermarket	(4, 75)	49	15
		Other Food Outlet	(47, 1094)	838	315
Street-network buffer around home	2 km	Convenience store	(2, 95)	23	21
		Fast-food Outlet	(2, 165)	46	37
		Supermarket	(0, 11)	5	3
		Other Food Outlet	(3, 578)	62	83
	4 km	Convenience store	(3, 262)	87	60
		Fast-food Outlet	(3, 460)	163	109
		Supermarket	(0, 33)	18	9
		Other Food Outlet	(11, 848)	253	244
	6 km	Convenience store	(15, 347)	194	95
		Fast-food Outlet	(14, 599)	363	168
		Supermarket	(1, 62)	37	15
		Other Food Outlet	(21, 1021)	633	348
Street-network Buffer around workplace	2 km	Convenience store	(2, 101)	34	33
		Fast-food Outlet	(3, 185)	69	58
		Supermarket	(1, 12)	4	3
		Other Food Outlet	(5, 597)	176	222
	4 km	Convenience store	(3, 260)	130	82
		Fast-food Outlet	(6, 464)	249	137
		Supermarket	(3, 39)	21	9
		Other Food Outlet	(22, 923)	462	340
	6 km	Convenience store	(22, 363)	246	85
		Fast-food Outlet	(52, 668)	452	141
		Supermarket	(10, 66)	45	12
		Other Food Outlet	(110, 1136)	839	284
Circular buffer around home and workplace	2 km	Convenience store	(20, 176)	90	45
		Fast-food Outlet	(25, 318)	173	81
		Supermarket	(5, 24)	15	4
		Other Food Outlet	(59, 734)	329	257
	4 km	Convenience store	(105, 384)	259	74
		Fast-food Outlet	(25, 318)	173	81
		Supermarket	(26, 70)	49	11
		Other Food Outlet	(240, 1176)	813	266
	6 km	Convenience store	(259, 749)	406	80
		Fast-food Outlet	(272, 1074)	430	117
		Supermarket	(51, 154)	79	17
		Other Food Outlet	(636, 2275)	1232	221
Straight-line buffer	2 km	Convenience store	(23, 290)	122	65
		Fast-food Outlet	(32, 528)	235	116
		Supermarket	(6, 54)	22	9
		Other Food Outlet	(64, 955)	422	286
	4 km	Convenience store	(109, 468)	279	86
		Fast-food Outlet	(180, 834)	526	145
		Supermarket	(27, 96)	53	15
		Other Food Outlet	(245, 1355)	866	284
	6 km	Convenience store	(260, 615)	414	77
		Fast-food Outlet	(471, 1169)	762	150
		Supermarket	(51, 126)	81	17
		Other Food Outlet	(676, 1911)	1360	237

Table 7 shows that in all RDFE there was a decrease in PPV as buffer size increased. In 2 km CBH for instance, the PPV for all food outlets were ≥ 0.75 which dropped to > 0.5 and ≤ 0.5 with 4 km and 6 km buffers, respectively. Of all RDFE, SNBH consistently showed the highest PPV, which again decreased with an increase in buffer size. For example, 2 km buffer had an the highest PPV (CS [0.94], fast-food outlet [0.93], other [0.95], and supermarket [1]), 4 km had moderately good PPV (CS [0.66], fast-food outlet [0.69], other [0.77] and supermarket [0.61]) and 6 km buffer had low PPV (CS [0.48], fast-food outlet [0.51], other [0.58], supermarket [0.36]). In contrast, the sensitivity of RDFE increased as buffer sizes increased. For instance, PPV for SNBH increased slightly from very low (≈0.1) in 2 km to > 0.3 in 4 km and > 0.58 in 6 km buffers. Similar patterns were observed across all RDFE. A buffer size of 4 km seemed to have a moderately good PPV (≈ 0.5 or above) for all RDFE.

**Table 7:** Mean food outlet count, Positive Predictive Value and Sensitivity of different RDFE

RDFE		Food outlet type	TP	FN	FP	Total Food outlet		PPV	95% CI	Sens	95%CI
Type	Size					AS	RDFE				
Circular Buffer around Home	2 km	Convenience store	48	183	10	231	58	0.83	[0.78, 0.88]	0.29	[0.23, 0.36]
		Fast-food outlet	62	237	12	299	74	0.86	[0.82, 0.90]	0.28	[0.21, 0.35]
		Other	91	530	12	621	103	0.87	[0.81, 0.91]	0.23	[0.16, 0.30]
		Supermarket	6	21	2	27	8	0.75	[0.66, 0.83]	0.32	[0.25, 0.39]
	4 km	Convenience store	111	120	96	231	207	0.55	[0.49, 0.61]	0.57	[0.49, 0.64]
		Fast-food outlet	144	155	111	299	255	0.58	[0.52, 0.64]	0.55	[0.47, 0.63]
		Other	274	347	167	621	441	0.62	[0.55, 0.68]	0.51	[0.42, 0.60]
		Supermarket	14	13	13	27	27	0.53	[0.46, 0.60]	0.57	[0.49, 0.66]
	6 km	Convenience store	156	75	231	231	387	0.4	[0.36, 0.46]	0.75	[0.68, 0.82]
		Fast-food outlet	205	94	257	299	462	0.45	[0.40, 0.50]	0.74	[0.67, 0.82]
		Other	427	194	391	621	818	0.5	[0.44, 0.57]	0.73	[0.65, 0.81]
		Supermarket	18	9	29	27	47	0.4	[0.35, 0.45]	0.74	[0.66, 0.81]
Circular Buffer around Workplace	2 km	Convenience store	61	170	18	231	79	0.72	[0.67, 0.77]	0.31	[0.25, 0.37]
		Fast-food outlet	91	208	20	299	111	0.78	[0.73, 0.82]	0.35	[0.29, 0.41]
		Other	231	390	25	621	256	0.79	[0.74, 0.85]	0.4	[0.32, 0.49]
		Supermarket	6	21	2	27	8	0.7	[0.62, 0.77]	0.31	[0.25, 0.37]
	4 km	Convenience store	126	105	150	231	276	0.44	[0.39, 0.49]	0.61	[0.54, 0.68]
		Fast-food outlet	177	122	183	299	360	0.48	[0.42, 0.53]	0.63	[0.56, 0.70]
		Other	376	245	222	621	598	0.55	[0.48, 0.61]	0.62	[0.54, 0.70]
		Supermarket	14	13	20	27	34	0.4	[0.35, 0.45]	0.59	[0.52, 0.65]
	6 km	Convenience store	169	62	292	231	461	0.37	[0.32, 0.42]	0.79	[0.72, 0.85]
		Fast-food outlet	228	71	334	299	562	0.41	[0.36, 0.46]	0.8	[0.74, 0.86]
		Other	488	133	504	621	992	0.63	[0.56, 0.69]	0.8	[0.73, 0.87]
		Supermarket	20	7	39	27	59	0.34	[0.30, 0.38]	0.79	[0.73, 0.86]
Circular Buffer around Home and Workplace	2 km	Convenience store	100	131	28	231	128	0.75	[0.71, 0.80]	0.5	[0.44, 0.56]
		Fast-food outlet	138	161	31	299	169	0.78	[0.74, 0.83]	0.36	[0.32, 0.39]
		Other	299	321	38	621	337	0.82	[0.77, 0.86]	0.52	[0.44, 0.60]
		Supermarket	11	16	4	27	15	0.74	[0.69, 0.79]	0.5	[0.43, 0.57]
	4 km	Convenience store	177	54	199	231	376	0.46	[0.41, 0.51]	0.82	[0.77, 0.86]
		Fast-food outlet	236	63	230	299	466	0.48	[0.42, 0.53]	0.82	[0.77, 0.87]
		Other	493	128	317	621	810	0.57	[0.51, 0.64]	0.81	[0.76, 0.87]
		Supermarket	20	7	26	27	46	0.43	[0.38, 0.48]	0.79	[0.74, 0.85]
	6 km	Convenience store	208	23	375	231	583	0.35	[0.31, 0.40]	0.93	[0.10, 0.96]
		Fast-food outlet	276	23	417	299	693	0.4	[0.35, 0.44]	0.94	[0.92, 0.96]
		Other	578	43	615	621	1193	0.48	[0.42, 0.55]	0.94	[0.92, 0.96]
		Supermarket	24	3	62	27	86	0.33	[0.29, 0.37]	0.93	[0.90, 0.96]
Street-network buffer around home	2 km	Convenience store	31	200	2	231	33	0.94	[0.92, 0.97]	0.19	[0.14, 0.24]
		Fast-food outlet	42	257	3	299	45	0.95	[0.92, 0.97]	0.19	[0.14, 0.25]
		Other	58	563	3	621	61	0.95	[0.93, 0.98]	0.16	[0.09, 0.22]
		Supermarket	4	23	0	27	4	0.79	[0.69, 0.89]	0.2	[0.16, 0.25]
	4 km	Convenience store	86	145	45	231	131	0.68	[0.63, 0.74]	0.46	[0.38, 0.54]
		Fast-food outlet	111	188	51	299	162	0.7	[0.64, 0.75]	0.44	[0.36, 0.52]
		Other	204	417	61	621	265	0.73	[0.67, 0.78]	0.4	[0.31, 0.49]
		Supermarket	11	16	7	27	18	0.61	[0.43, 0.55]	0.47	[0.39, 0.54]
	6 km	Convenience store	135	96	149	231	284	0.53	[0.48, 0.59]	0.67	[0.59, 0.74]
		Fast-food outlet	177	121	169	299	346	0.58	[0.51, 0.65]	0.66	[0.57, 0.74]
		Other	364	257	260	621	624	0.58	[0.51, 0.65]	0.65	[0.55, 0.74]
		Supermarket	16	11	29	27	45	0.48	[0.41, 0.54]	0.67	[0.59, 0.74]
Street-network buffer around workplace	2 km	Convenience store	39	192	6	231	45	0.89	[0.84, 0.94]	0.18	[0.14, 0.21]
		Fast-food outlet	63	236	6	299	69	0.91	[0.86, 0.95]	0.24	[0.19, 0.28]
		Other	177	444	7	621	184	0.93	[0.89, 0.96]	0.28	[0.21, 0.35]
		Supermarket	3	24	1	27	4	0.9	[0.84, 0.97]	0.14	[0.11, 0.17]
	4 km	Convenience store	103	128	86	231	189	0.53	[0.47, 0.59]	0.5	[0.43, 0.56]
		Fast-food outlet	147	152	103	299	250	0.57	[0.52, 0.63]	0.53	[0.46, 0.60]
		Other	329	292	123	621	452	0.65	[0.59, 0.71]	0.54	[0.46, 0.63]
		Supermarket	11	16	11	27	22	0.51	[0.45, 0.56]	0.59	[0.51, 0.65]
	6 km	Convenience store	153	78	208	231	361	0.41	[0.36, 0.46]	0.73	[0.66, 0.80]
		Fast-food outlet	209	90	245	299	454	0.45	[0.40, 0.50]	0.74	[0.66, 0.80]
		Other	457	164	391	621	848	0.51	[0.44, 0.59]	0.75	[0.68, 0.82]
		Supermarket	17	10	28	27	45	0.38	[0.34, 0.42]	0.72	[0.65, 0.78]
Straight-line Buffer	2 km	Convenience store	133	98	47	231	180	0.72	[0.67, 0.77]	0.62	[0.55, 0.68]
		Fast-food outlet	175	123	54	299	229	0.75	[0.70, 0.80]	0.63	[0.56, 0.70]
		Other	368	253	68	621	436	0.8	[0.75, 0.85]	0.62	[0.54, 0.70]
		Supermarket	15	12	6	27	21	0.71	[0.65, 0.77]	0.62	[0.56, 0.69]
	4 km	Convenience store	190	41	224	231	414	0.45	[0.40, 0.49]	0.85	[0.80, 0.89]
		Fast-food outlet	249	50	258	299	507	0.48	[0.43, 0.53]	0.85	[0.80, 0.89]
		Other	517	104	355	621	872	0.56	[0.50, 0.63]	0.84	[0.78, 0.89]
		Supermarket	21	6	30	27	51	0.41	[0.37, 0.46]	0.83	[0.78, 0.88]
	6 km	Convenience store	215	16	388	231	603	0.35	[0.31, 0.39]	0.94	[0.92, 0.92]
		Fast-food outlet	281	18	427	299	708	0.39	[0.35, 0.44]	0.95	[0.93, 0.97]
		Other	589	31	626	621	1215	0.48	[0.42, 0.54]	0.95	[0.92, 0.97]
		Supermarket	25	2	50	27	75	0.33	[0.29, 0.36]	0.94	[0.92, 0.97]
Standard Deviational Ellipse		Convenience store	153	78	140	231	293	0.6	[0.53, 0.67]	0.62	[0.54, 0.71]
		Fast-food outlet	193	106	152	299	345	0.63	[0.56, 0.70]	0.62	[0.54, 0.71]
		Other	384	237	216	621	600	0.67	[0.60, 0.75]	0.6	[0.51, 0.68]
		Supermarket	18	9	17	27	35	0.6	[0.52, 0.68]	0.6	[0.52, 0.69]

*TP*  True positive (food outlets in AS and RDFE), *FN* False Negative (food outlets in AS only); *FP*  False Positive (food outlets in RDFE only); *PPV*  Positive Predictive Value, *Sens  *Sensitivity, *RDFE*  Researcher-Defined Food Environment

### Aim 4: Explore whether exposure to food outlet reflects the use of food outlets.

A total of 250 food outlets visitations were made during the study period of which more than half (54%) were supermarkets. Convenience store (16%) and fast-food outlets (9%) were the outlets least visited. In contrast, participants had the highest exposure to ‘other’ food outlets in their AS (53%) followed by fast-food outlets (25%) and the least exposure was to supermarkets (2%) (Table 8).

**Table 8:** Exposure and the use of food outlets

Food outlet	Exposure n (%)	Use n (%)
Fast-food outlet	302 (25)	23 (9)
Convenience store	234 (20)	40 (16)
Supermarket	27 (2)	134 (54)
Other	632 (53)	29 (21)
Total	1,195 (100)	250 (100)

## Discussion

The findings of this study revealed that AS size was directly related to the distance from home to workplace. These findings agree with the study by Drewnowski et al*.* [15] which found that GPS-based AS size had a positive association with distance from home to work. Our study also showed that the orientation of AS for most of the participants working away from home followed the home-to-workplace direction, which signifies the importance of the two locations in an individual movement. These findings align with the findings of studies on spatial analysis of the AS in which most of the activities of participants were undertaken around activity anchor points [22, 33, 57]. Saxena and Mokhtarian [50] also found that most activities of telecommuter (individuals who work from home for an organisation) were carried out around home on telecommuting days while most of the destinations were oriented toward the workplaces on commuting days. These findings have an important implication during and post COVID 19 when working from home has and may become more common [3, 55]. Researchers need to consider the change in individuals AS patterns considering where individuals spend most of their day when modelling their AS.

It was also found in this study that younger participants aged less than 30 years had smaller activity space compared to those aged 41–50 years. Similarly, the study revealed that the manager and the professional occupational group had larger activity space compared to the manual and routine worker group. This could be influenced by the fact that most of the younger participants were students who lived near their workplaces while the older participants were senior employees who lived further from their workplaces meaning that participants younger than 30 years could have a shorter commute than the older participants.

The study also found a positive correlation between the participants’ mode of transport and the AS in which participants using active transport (e.g. walking or cycling) had smaller AS compared to those using passive transport (e.g. car and bus). Similar findings were reported in the study by Zenk et al. [65]. Despite differences in activity space size among different age, income occupation groups and mode of transport, the study did not find any association between the size of activity space and other social demographic characteristics and SES. Similar findings were observed in a study by Drewnowski et al. [15] which found no association between the size of AS and other social demographic characteristics and SES. Similar findings were observed in a study by Drewnowski et al. [15] which found no statistically significant relationships between the participants' sociodemographic characteristics and the areal size of their AS.

Literature suggests that AS provides a more realistic representation of individual exposures to food outlets compared to researcher-defined measures such as buffers [8, 21, 65]. When RDFE was superimposed on AS—a reference measure of “actual” exposure—to determine the percentage overlap of the two, all RDFE misrepresented the AS and the percentage mean overlap between the RDFE and AS varied considerably across different RDFE type and size. These findings agree with a study by Sadler and Gilliland [48] on 526 children using a GIS-based analysis of individuals’ GPS tracks AS and different proxies for AS like buffers and container approaches to quantify the discrepancies resulting from the use of different proxy methods. The study showed that exposure proxies consistently underestimate exposure to junk foods by up to 68%.

The analysis also showed that the RDFE which included both home and workplace such as SLB and CBHW had a better representation (overlap) of AS compared to those which considered home (i.e. SNBH and CHB) or workplace (SNBW and CHW) separately. Thus, the use of RDFE which focuses on home or workplace separately has potential for errors in estimating AS unless both home and workplace are considered. Here state how much and give examples of studies that have done this and the spurious conclusions they have made.

In future research where it is not possible to capture individual AS using GPS data, home, workplace and commute could be used to model individuals’ AS. The current study suggests a 4 km buffer may provide a fair representation of exposure, however, further research is needed to establish an optimum buffer size that captures exposure for different populations.

Furthermore, the study confirmed that commonly used measures of the food environments (i.e. RDFE) lack precision and accuracy representing exposure. PPV for all RDFE were inversely related to the size of the RDFE. Although smaller buffers had a high probability of capturing food outlets that were truly present in the AS, they missed a considerable number of food outlets in the AS, therefore underestimating exposure. Conversely, although the larger RDFE captured more food outlets, they significantly included both non-AS and many false-positive food outlets, leading to a lowering of their PPV.

**Fig. 4:**
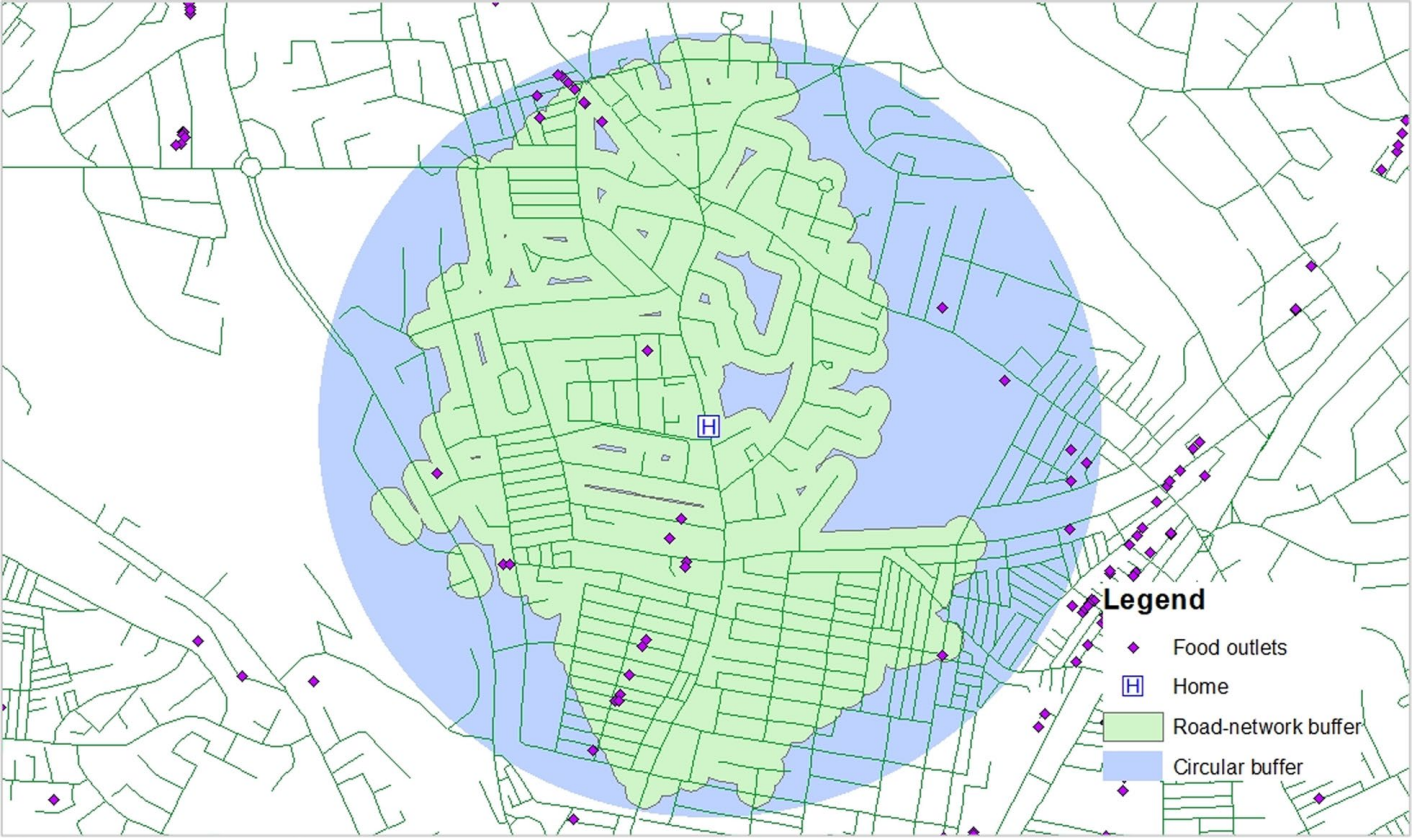
An example showing a 2 km circular buffer around home covering wider and non-activity space than a 2 km street-network buffer

The type of RDFE also mattered when it came to the PPV of the RDFE. The street network buffers, for instance, had slightly higher PPV compared to circular buffers. Street-network buffers may more closely reflect the on-the-ground context; by excluding non-activity areas they reduce false-positive food outlets (Fig. 4) [5, 26, 41]. Circular buffers are considered to be less representative of the “actual” relevant spatial context of places, especially in locations with natural features like water bodies or other features like railways [41]. Yet, circular buffers remain the most commonly used buffers in food environment research [32, 63].

The inclusion of both home and workplace in the RDFE (e.g. CBHW and SLB) significantly improved the sensitivity of RDFE, but not the PPV. This supports a huge body of literature indicating powerful exposures beyond home neighbourhoods [11].

In quantifying how much the RDFE insufficiently represents exposure in the food environment these findings have significant implications. Policymakers need to interpret existing evidence with caution when making decisions about modifying food environments. Meanwhile, researchers in the food environment field ought to be aware of how much exposure is missed out and traded-off when using certain types or sizes of RDFE. Future studies should include both home and workplace locations to improve the representation of the food environment and exposure to food outlets.

Despite high exposure to ‘other’ and ‘fast-food’ outlets, participants in this study used these food venues relatively rarely; most food purchases were made at supermarkets, whereas they made the fewest food purchases from fast-food outlets. These findings align with Appelhans et al. [1] who found supermarket stores to be the most visited food outlet while fast-food/takeaway outlets were the least visited food venue. The analysis also revealed that exposure to food outlet was not related to their use. This suggests that mere exposure to a certain type of food outlet may not necessarily lead to the use of those facilities [16]. The mechanism by which exposure influences use is likely to be more complex than has been suggested by most contemporary research. Glanz et al. [19] for instance highlighted taste, cost, convenience, variety and energy density as some of the key determinants of food choices. These factors may be objective, subjective or both, powerfully influencing individuals’ food choices and purchase locations.

Several studies in the food environment suggest that exposure to certain types of food outlets increases the likelihood of adiposity [4, 6, 7, 30]. These studies operate under the assumption that some food outlets are ‘healthy’ while others are ‘unhealthy’ [20]. Often, fast-food outlets, takeaways and convenience stores are classified as ‘unhealthy’, whereas grocery stores and supermarkets are considered ‘healthy’ [20]. This stratification of food outlets is overly simplistic and problematic, it fails to recognise the wide array of unhealthy food options offered within most supermarkets while disregarding the healthy food options available at most fast-food outlets [20]. A study by Lesser et al. [34] for instance, demonstrated that the average amount of calories purchased by participants at McDonald's (1,038 cal)—considered as ‘unhealthy’—was the same as the calories purchased at Subway (955 cal)—considered as ‘healthy’. The so-called ‘health halo’ [14, 34] can spuriously imply that certain specific food outlets are ‘healthier’. Importantly, the ‘healthiness’ of food outlets is determined by the actual food offered by the food outlets [20].

It is important to consider that a limitation of the current study is the small and non-representative sample (i.e. majority of the participants were female). Although these findings are not generalisable, the study managed to collect the quality and rich individual-level data that permitted exploration of the food environment using a wide range of buffer types and sizes. Coupled with multiple food environment measures, this distinctive combination of variables offered valuable comparisons between these metrics and added insights on the discrepancies existing in the RDFE measures. Relying on a cross-sectional design also limits the causality that may be drawn from the findings. Even allowing for this shortcoming, the findings justify serious consideration on the effects of using different measures of food environments on the exposure in the food environment.

## Conclusion

This study has highlighted the importance of home and workplace locations in the food environments and the limitations of the RDFE in representing an individual’s food environments and exposure to food outlets. Different RDFE had varying accuracy of representing exposure in the food environment. Fundamentally, the majority of RDFE misrepresent exposure. Therefore, it is advisable for researchers using datasets without individual mobility information—GPS tracking (e.g., secondary data) to consider including both home and workplace for individuals working away from home when modelling AS or food environments in their analysis. Over-dependence on conventional ways (proxy measures) of measuring exposure, which are obviously incomplete, proposes an under-developed appreciation of how the environment influences weight status. Moreover, exposure to food outlets was not a good determinant of their use. Clearly, the relationship between exposure and use is more complex than is currently suggested in both empirical and policy literature. With an increased impetus to modify the food environment, policymakers ought to be cautious when interpreting the current evidence in this field which is greatly based on the RDFE. We suggest a shift of focus in the food environment field from mere exposure to food outlets to more nuanced factors like the quality of food offerings by food outlets and the quality and quantity of food purchased and consumed by people.

## Data Availability

The data that support the findings of this study are available from Leeds Beckett University, but restrictions apply to the availability of these data, which were used under license for the current study, and so are not publicly available. Data are however available from the authors upon reasonable request and with permission of Leeds Beckett University.
